# *IRF5* gene polymorphisms in melanoma

**DOI:** 10.1186/1479-5876-10-170

**Published:** 2012-08-21

**Authors:** Lorenzo Uccellini, Valeria De Giorgi, Yingdong Zhao, Barbara Tumaini, Narnygerel Erdenebileg, Mark E Dudley, Sara Tomei, Davide Bedognetti, Maria Libera Ascierto, Qiuzhen Liu, Richard Simon, Leah Kottyan, Kenneth M Kaufman, John B Harley, Ena Wang, Steven A Rosenberg, Francesco M Marincola

**Affiliations:** 1 Infectious Disease and Immunogenetics Section (IDIS), Department of Transfusion Medicine, Clinical Center and trans-NIH Center for Human Immunology, National Institutes of Health, Bethesda, MD 20892, USA; 2 Institutes of Infectious and Tropical Diseases, University of Milan, L Sacco Hospital, Milan, Italy; 3 Biometric Research Branch, Division of Cancer Treatment and Diagnosis, National Cancer Institute, National Institutes of Health, Bethesda, MD 20892, USA; 4 Cell Processing Section, Department of Transfusion Medicine, Clinical Center, National Institutes of Health, Bethesda, MD 20892, USA; 5 Department of Internal Medicine (DiMI), University of Genoa, Genoa 16132, Italy; 6 Center for Autoimmune Genomics and Etiology (CAGE), Cincinnati Children’s Hospital Medical Center, Cincinnati, OH 45229, USA; 7 U S Department of Veterans Affairs Medical Center, Cincinnati, OH 45229, USA; 8 Surgery Branch, National Cancer Institute, National Institutes of Health, Bethesda, MD, USA

## Abstract

**Background:**

Interferon regulatory factor (*IRF)-5* is a transcription factor involved in type I interferon signaling whose germ line variants have been associated with autoimmune pathogenesis. Since relationships have been observed between development of autoimmunity and responsiveness of melanoma to several types of immunotherapy, we tested whether polymorphisms of *IRF5* are associated with responsiveness of melanoma to adoptive therapy with tumor infiltrating lymphocytes (TILs).

**Methods:**

140 TILs were genotyped for four single nucleotide polymorphisms (rs10954213, rs11770589, rs6953165, rs2004640) and one insertion-deletion in the *IRF5* gene by sequencing. Gene-expression profile of the TILs, 112 parental melanoma metastases (MM) and 9 cell lines derived from some metastases were assessed by Affymetrix Human Gene ST 1.0 array.

**Results:**

Lack of A allele in rs10954213 (G > A) was associated with non-response (p < 0.005). Other polymorphisms in strong linkage disequilibrium with rs10954213 demonstrated similar trends. Genes differentially expressed *in vitro* between cell lines carrying or not the A allele could be applied to the transcriptional profile of 112 melanoma metastases to predict their responsiveness to therapy, suggesting that *IRF5* genotype may influence immune responsiveness by affecting the intrinsic biology of melanoma.

**Conclusions:**

This study is the first to analyze associations between melanoma immune responsiveness and *IRF5* polymorphism. The results support a common genetic basis which may underline the development of autoimmunity and melanoma immune responsiveness.

## Introduction

The development of autoimmunity in patient with malignant melanoma has been linked to tumor regression following immunotherapy with interleukin (IL)-2, interferon (IFN)α or anti-Cytotoxic T-Lymphocyte Antigen (CTLA)-4. Examples include hypothyroidism [1,2], *vitiligo*[3], anti-phospholipids syndrome [4] and autoimmune retinopathy [5]. In particular, the fact that effective immune responses against melanoma are associated with the development of *vitiligo*[3], which results from autoimmune destruction of normal melanocytes, reinforces the pathogenic commonality between autoimmunity and cancer rejection.

Several studies support the hypothesis that distinct immune-mediated tissue-specific destruction (TSD) processes such as autoimmunity, cancer rejection, allograft rejection, graft versus host disease, or acute infection resulting in clearance of pathogens share convergent final mechanisms [6-9]; we have previously defined this phenomenon as the immunological constant of rejection (ICR) [6]. The ICR includes the coordinate expression of genes controlling antigen presentation, interferon-stimulated genes (ISGs) and immune effectors functions (IEFs). The suggestion that the phenomena of immune-mediated cancer rejection and autoimmunity represent different faces of TSD, leads to the conjecture that factors responsible for autoimmunity might also be relevant to the immunotherapy of cancer.

Systemic Lupus Erythematosus (SLE) is the prototype for systemic autoimmune disease. An important step to understand the pathogenesis of SLE was the appreciation for the dominant role played by type I IFNs [10,11]. Variants of genes within the IFN pathway like interferon regulatory factor (*IRF)-5* have been associated with SLE by multiple studies [12,13] and have been repeatedly implicated in susceptibility to several other autoimmune diseases including rheumatoid arthritis [14], multiple sclerosis [15,16], inflammatory bowel disease [17] and Sjogren’s syndrome [12,18]. *IRF5* is involved in host defense against pathogens by inducing transcription of IFNα [19] and the expression of genes involved in apoptosis [20,21]. Three functional variants of *IRF5* that are associated with SLE risk have been touted to define the risk to develop SLE: including a splice site, a 30 base pair in-frame insertion/deletion, and an alternative polyadenylation site in the 3’UTR region [22]; however, no fine mapping study has been reported to establish whether these, over the many other variants, including an interesting promoter variant [23] are preferred as explanations for SLE risk.

The prominent role played by *IRF5* variants in determining the risk to develop autoimmunity suggests a possible role for *IRF5* as modulator of immune responsiveness of melanoma. However, to our knowledge, *IRF5* polymorphisms have never been studied in the context of melanoma. Therefore, we investigated whether polymorphisms in *IRF5* associated to SLE are also associated with melanoma responsiveness to immunotherapy. Concordant to results obtained in SLE, the lack of the A allele in rs10954213 (G > A) that is protective against the development of SLE was associated to non-responsiveness to treatment among 140 patients with metastatic melanoma who received the adoptive transfer of tumor infiltrating lymphocytes (TILs). Remarkably, transcriptional changes observed between melanoma cell lines carrying or not the A allele could be used to predict responsiveness of 112 melanoma metastases (MM), suggesting that the *IRF5*-dependent immune responsiveness is at least partly related to the intrinsic biology of melanoma.

## Materials and methods

### Patient samples

TILs were expanded *in vitro* from 140 excised melanoma metastases for reinfusion into patients following lympho-depletion of the host. An aliquot from each TIL preparation were cryo preserved on the day of infusion. Samples were collected during 5 consecutive trials at the Surgery Branch, National Cancer Institute (NCI) [24,25]. Before TIL administration, patients received nonmyeloablative lymphodepletion consisting of cyclophosphamide at 60 mg/Kg/d for 2 days and fludarabine at 25 mg/m2/d for 5 days [24]. Two Gy or 12 Gy total body irradiation (TBI) was administered in conjunction with chemotherapy in T200 and T1200 trials, respectively. Within 1 day of completion of lymphodepletion, TILs were infused and high-dose IL-2 therapy was started (720,000 IU/Kg intravenously every 8 hours to tolerance). Two days after TIL infusion, patients treated with TBI also received autologous purified CD34+ hematopoietic stem cells from a granulocyte colony-stimulating factor ± plerixafor.

Different protocols were employed to generate TILs [24-26]. A ‘classic’ method employing an extended duration of multiple microcultures and an individualized assay to identify tumor recognition was used for TNMA, T200, T1200 trials [24,26]. A simplified method using short-term cultured ‘young’ TILs unscreened for tumor-reactivity were used in TYT and TCD8 trials [25]. TILs were depleted from CD4+ cells in TCD8 trial [25].

Inclusion criteria for the trials were: age ≥ 18, measurable disease, good clinical performance and a life expectancy above 3 months. All patients signed an informed consent approved by the NCI Institutional Review Board. Data for this analysis are updated as of January 11^th^, 2012. Response (complete response CR, partial response PR or no response NR) was assessed using the Response Evaluation Criteria in Solid Tumors (RECIST) guidelines starting approximately 4 weeks after TIL administration and at regular intervals thereafter. A CR or PR was considered an overall response (R).

TIL samples from 142 melanoma patients under adoptive transfer therapy were available: RNA and DNA were isolated from the same 140 TIL with 2 additional TIL for RNA only. 112 pre-treatment snap-frozen tumor biopsies used for the TIL generation were used for RNA extraction. RNA and DNA were also isolated from 15 melanoma cell lines derived from the 112 melanoma lesions.

### Genotyping

Four single nucleotide polymorphisms (SNPs) rs10954213, rs11770589, rs6953165, rs2004640 and one insertion-deletion in exon 6 of *IRF5* were genotyped by sequencing 140 TILs. PCR was carried out in a reaction mixture containing 40 ng of DNA, 10 μl of HotStar Taq Master Mix (Qiagen, Germantown, MD) and 100 pmol of each of the following primers: for rs10954213/rs11770589, forward 5-CCCTGATTTCCCTGGTTTG-3 and reverse 5’-AGCCAGCCAGGTGAGTGTT-3’; for rs6953165/rs2004640 forward 5’-CACCGCAGACAGGTGGG-3’, reverse 5’-GGGAGGCGCTTTGGAAGT-3’; for insertion-deletion in exon 6 forward 5’-CCCCACATGACACCCTATTC-3’ and reverse 5’-GGCTGGGGTCTGGAGCAG-3’. The reaction mixture was denatured at 95°C for 15 minutes and cycled 35 times at 94°C for 45 seconds, T_a_ for 45 seconds (T_a_ = 55°C for rs10954213 / rs11770589; T_a_ = 56°C for rs6953165 / rs2004640; T_a_ = 58°C for insertion-deletion), 72°C for 60 seconds, with final extension at 72°C for 10 minutes. PCR product was treated with Exosap-IT (USB Corporation, Cleveland, OH) to removed excess primers. 3.5 μl out of 20 of purified DNA product was combined with 2.0 μl of Big Dye terminator (ABI Prism Big Dye Terminator cycle sequencing ready reaction kit v3.1, Applied Biosystems, Foster City, CA) and 100 pmol of forward primer. The sequencing reaction was carried out for 30 cycles of denaturation (96°C/1 m), annealing (50°C/30 s) and extension (60°C/ 4 m). Excess dye terminators were removed using DyeEx 96 Kit columns as per Manufacturer’s instructions (Qiagen Inc. Germantown, MD). Electrophoresis was performed on ABI Prism 3730 XL instrument (Applied Biosystems, Foster City, CA).

### Statistical analysis

In this explorative analysis, no stratification of patients was done according to distinct TIL protocols with the assumption that the genetic background of the patient would have an independent effect on responsiveness to the conceptually similar therapeutic protocols studied here. Association analyses were conducted by chi-square test using 2 × 2 and 2 × 3 contingency tables. When 1 or more variables in the contingency table were ≤ 5, Fisher’s exact test was used. Two side probability values (p_2_-value) < 0.05 were considered to be statistically significant. In gene expression data analysis, the variance of the log-intensity for each gene was compared to the median of all the variances. Those genes not significantly more variable than the median gene were filtered out (p > 0.01). For class comparison in cell lines with replicates, a linear mixed-effects model was used for each gene (implemented in BRB-ArrayTools). Cell line was specified as random effect and IFNα and genotype were specified as fixed factors. Genes that were differentially expressed between genotype “A” and “G” groups were identified after accounting for the differences in expression in IFNα + and IFNα- groups. A list of genes whose expression changes due to IFNα were also identified by adjusting for any imbalances in the “A” to “G” ratio in the IFNα + and IFNα- groups. To confirm the segregation of two distinct groups in class comparison analysis data, a K-means clustering algorithm was performed that chooses a pre-specified number of cluster centers to minimize the within class sum of squares from those centers [27].

Calculation of linkage disequilibrium (LD) parameters (r^2^ and D’) based on genotype data from 140 patients was performed using LDPlotter tool from https://pharmgat.org/Tools.

### Gene expression profiling

We generated 3 independent gene expression data sets from 140 TIL samples, 112 melanoma metastases and 9 cell lines out of 15 that were homozygous for a given *IRF5* haplotype and had been expanded from some of the metastases. Total RNA was extracted using miRNeasy minikit (Qiagen, Germantown, MD). The cell lines were treated with 1000 U*/*mL IFNα2b and RNA was isolated after 9 hours. RNA quality and quantity was estimated using Nanodrop (Thermo Scientific, Wilmington, DE) and Agilent 2100 Bioanalyzer (Agilent Technologies, Palo Alto, CA). RNA was amplified from 300 ng of total RNA (Ambion WT Expression Kit). cDNA was reverse transcribed with biotinilation and hybridized to the GeneChip Human Gene 1.0 ST Arrays (Affymetrix WT Terminal Labeling Kit,) after fragmentation. The arrays were washed and stained on a GeneChip Fluidics Station 450 (Affymetrix, Santa Clara, CA); scanning was carried out with the GeneChip Scanner 3000 and image analysis with the Affymetrix GeneChip Command Console Scan Control. Expression data were normalized using the RMA algorithm, http://www.partek.com (Partek Inc., St. Louis, MO and log-transformed (base 2) for parametric analysis and cluster analysis was based on Partek software. Functional interpretations were based on Ingenuity Pathways Analysis software ( http://www.ingenuity.com (Ingenuity Systems, Inc., Redwood City, CA).

## Results

### Genotyping results

**Figure 1:**
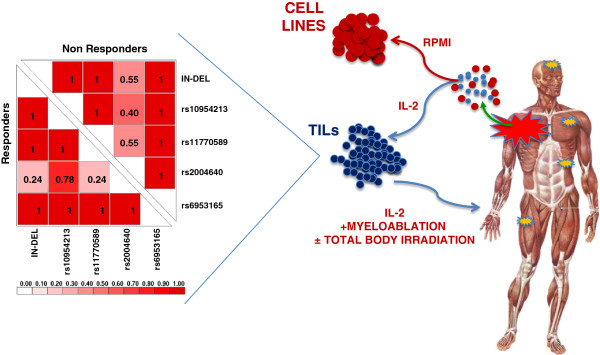
**(Left) Pairwise linkage disequilibrium between *IRF5 *polymorphisms in tumor infiltrating lymphocytes (TILs).** D’ and r^2^ were calculated using LDPlotter tool. D’ values for linkage disequilibrium among the major alleles of each of *IRF5* are shown. IN-DEL = insertion/deletion. (Right) Samples studied: 140 TILs, 112 parental melanoma metastases and 9 cell lines derived from some metastases.

*Genotyping of TILs* – Sequencing germline DNA extracted from 140 TILs for *IRF5* rs10954213, rs11770589 and rs6953165, rs2004640 and one insertion-deletion in exon 6 demonstrated that genotype frequency distribution was significantly different between responders and non-responders (Table 1). All *IRF5* variants but rs2004640 were associated with immune responsiveness. The lack of the A allele in rs10954213 was predominant in non-responders (rs10954213: AA + AG: 63R vs. 50NR, GG: 7R vs. 20NR, P = 0.005), (Table 2). All investigated polymorphisms but rs2004640 are in LD with each other in both responders and non-responders (r^2^ = 1, D’ = 1, Figure 1). Because of the strong linkage disequilibrium among the different variants, we selected one (rs10954213) that provided the highest predictive value for subsequent class comparison analyses as representative of the *IRF5* extended haplotype. Moreover, because the presence of the A allele in this SNP was associated with immune responsiveness independent of homo/hetorozygosity, we split samples into an “A” allele carrier (AA + AG) and an “A” allele missing (GG) group.

**Table 1:** IRF5 genotype frequencies

	**Genotype**	**R**	**NR**	**#total**	**P**
rs10954213	AA	33 (63.5)	19 (36.5)	52 (37.1)	0.007
	AG	30 (49.2)	31 (50.8)	61(43.6)	
	GG	7 (25.9)	20 (74.1)	27 (19.3)	
rs1177589	AA	18 (60)	12 (40)	30 (21.4)	0.012
	AG	39 (57.4)	29 (42.6)	68 (48.6)	
	GG	13 (31)	29 (69)	42 (30)	
rs2004640	GG	16 (43.2)	21 (56.8)	37 (26.5)	0.6219
	GT	34 (53.2)	30 (46.8)	64 (45.7)	
	TT	20 (51.2)	19 (48.8)	39 (27.8)	
rs6953165	CC	66 (60)	57 (40)	123 (87.9)	0.059
	CG	4 (57.4)	12 (42.6)	16 (11.4)	
	GG	0 (31)	1 (69)	1 (0.7)	
exon6	del/del	18 (60)	12 (40)	30 (21.4)	0.011
	ins/del	39 (57.4)	29 (42.6)	68 (480.6)	
	ins/ins	13 (31)	29 (69)	42 (30)	

**Table 2:** IRF5 rs10954213-TILs association

**Genotype**	**R**	**NR**	**Total**	**P**
AA + AG	63 (55.8)	50 (44.2)	113 (81.0)	0.005
GG	7 (25.9)	20 (74.1)	27 (19.0)	
			140 (100)	

*Genotyping of cell lines* – all the 15 cell lines were genotyped for rs10954213 (G > A) polymorphism to test the degree in which this trait was conserved within the instable genetic background of cancer cells. All of the cell lines displayed the genetic profile predicted by the germline analysis with the exception of one (3025 Mel), which demonstrated loss of heterozygosis (LOH, loss of the A allele; AG > G genotype) (Table 3). This loss was significant because it leads to the absence of the dominant “A” allele determining immune responsiveness. For this reason, although the patient bore a mixed genotype, the hemi-zygotic “G” cell line was considered together with the “G” homozygous cell lines for class comparison. However, the patient and corresponding tumor were considered still heterozygous because there is no clear evidence that the cell line genotype was representative of the cancer cells *in vivo*, nor it was known whether the transcriptional profile of tumors containing a mixed population of cancer and normal cells would be predominantly affected by one or the other. In addition, it is not known whether the *IRF5* genotype of the tumor or some other cell type, such as an immune cell, is where the genotype is relevant to the immune-responsiveness phenotype. Certainly, if there an elevated proportion of patients have metastases showing loss of heterozygosity the “A” allele at *IRF5*, then this would imply that the *IRF5* genotype of the tumor itself is important. In any case, the frequency of LOH was < 10%. Thus, we estimated, for the purpose of further analysis, that the genotype of cancer cells in tumor tissues was conserved in approximately 90% of cases and conducted further class comparisons classifying metastases according to the genetic background of the corresponding patient.

**Table 3:** Fifteen melanoma cell lines genotype compared with the germline

**Cell lines ID**		**rs10954213 (G > A)**	
		**Melanoma cell lines**	**Germline**
TIL_120	3104	AA	AA
TIL_064	2458	AA	AA
TIL_121	3107	AA	AA
TIL_030	2155	AA	AA
TIL_077	2744	AA	AA
TIL_048	2492	AA	AA
TIL_047	2448	AA	AA
TIL_032	2224	AG	AG
TIL_062	2523	AG	AG
TIL_013	2035	AG	AG
TIL_040	2427	AG	AG
TIL_016	2075	AG	AG
TIL_109	3025	YG (LOH)^*^	AG
TIL_005	1866	GG	GG
TIL_088	2805	GG	GG

*LOH=Loss Of Heterozygosis.

### Gene expression profiling

#### Cell lines

**Figure 2:**
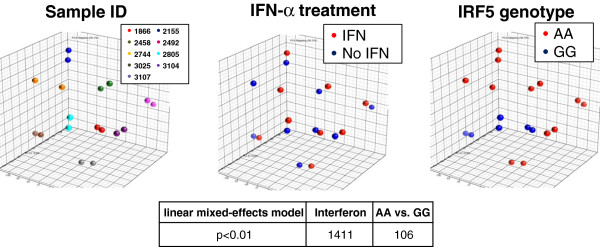
**(Top panel) Principal Component Analysis (PCA) based on the gene expression profile of 9 out of 15 melanoma cell lines.** The 3D displays of the PCA are color coded by sample ID, IFNα treatment, and *IRF5* genotype, respectively. (Bottom panel) Gene expression analysis of cell lines using linear mixed-effects model shows 106 genes were found significant (p < 0.01) for testing the genotype effect and 1411 genes were found significant (p < 0.01) for testing the interferon effect.

Nine of the 15 cell lines generated from the melanoma metastases were analyzed for functional differences according to their *IRF5* genotype; the genetic profile of these cell lines and its relationship with the parental tumors has been extensively described elsewhere [28]. To test whether differences between *IRF5* genotype bear any effects on the intrinsic biology of cancer cells without the influence of the microenvironment, we cultured the 9 cell lines with or without IFNα and compared transcriptional patterns according to their *IRF5* rs10954213 genotype. Seven cell lines out of the 15 bore the more frequent “AA” genotype but one of them stopped growing in culture, reducing to 6 the number of cell line analyzed; whereas three (including the “G” hemizygous cell line) bore only the GG allele. Principal Component Analysis (PCA) (Figure 2) demonstrated that identical cell lines distributed closely in three-dimensional spaces independently of the treatment with IFNα with the three G only carrying cell lines clustering close together. IFNα predicatively affected the expression of 1,411 genes (p < 0.01), mostly related to IFN signaling (see Additional file 1: Table S1). We identified 106 differentially expressed genes between “A” and “G” groups using a linear mixed-effects model (p < 0.01). Gene enrichment analysis suggested that the predominantly affected pathways were related to antigen presentation, immune response, allograft rejection, autoimmunity and metabolism.

#### Re-clustering of melanoma metastases based on transcripts differentiating melanoma cell lines with distinct IRF5 genotype

**Figure 3:**
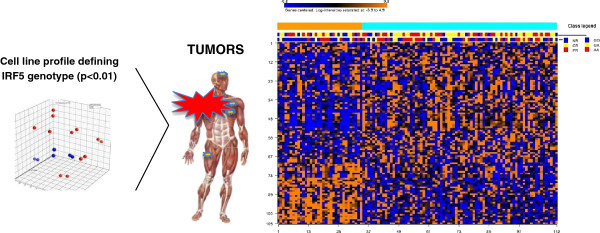
**Cell lines predict tumor behavior, comparing AA vs GG.** K mean clustering method organize heat map of melanoma metastases re-clustered based on 106 genes differentiating melanoma cell lines with distinct *IRF5* genotype. On the right side of the panel, a subset of metastases significantly enriched in non-responders; on the left side of the panel, a set of cases that enriched in responders (Fisher test: p_2_-value = 0.025).

The 106 gene signature differentiating melanoma cell lines *in vitro* was applied to produce a heat map of melanoma metastases based on K mean clustering method (Figure 3). This experiment identified a subset of metastases significantly enriched in non-responders. Interestingly, while this signature appeared to strongly predict lack of immune responsiveness, it was less predictive of the *IRF5* genotype of the patient, suggesting that the relationship between the two parameters is complex and multifactorial. K-means clustering algorithm, performed on the class comparison of metastases based on transcripts differentiating melanoma cell lines with distinct *IRF5* genotype, segregated two groups of patients. The group 2 includes 46 responders and 32 non-responders, while the group 1 includes 12 responders and 22 non-responders, with in this case inclusion of a large majority of GG genotypes (Fisher test: p_2_-value = 0.025).

## Discussion

Several studies support a link between autoimmunity and cancer immune responsiveness, suggesting that the two phenomena share a common determinism. Thus, we hypothesized that factors relevant to autoimmunity might also be relevant to cancer immunotherapy. The *IRF5* gene has been associated with SLE in multiple ethnic groups and repeatedly implicated in susceptibility to many autoimmune diseases, becoming a rationale for the focus of our study. Consistent with the concept that autoimmunity and cancer rejection might represent different facets of the same phenomenon, we observed that polymorphisms protecting against the susceptibility to develop SLE [12,13], such as the *IRF5* rs10954213 GG genotype, were significantly more prevalent among patients who did not respond to adoptive TIL therapy.

We analyzed cell lines grown from 9 metastases to test the weight of the *IRF5* genotype on the intrinsic biology of cancer cells independent of microenvironment influences. This allowed us to test the ability of the *IRF5* genotype to predict immune responsiveness *in vivo*. We applied the signatures of genes differentiating the 2 cell line genotypes *in vitro* to the melanoma metastases *in vivo* and observed a significant segregation of responders from non-responders, leading to the conclusion that immune responsiveness is at least in part dependent upon the genetic background of the host, which affects the biology of cancer cells primarily, and secondarily the immune responsiveness of tumors.

It is interesting to observe that the *IRF5* genotype appeared to segregate 2 different cases. When genes differentiating melanoma cell lines *in vitro* according to genotype were applied for class prediction, a segregation of responding and non responding cases was observed and it was only partially predictive of the *IRF5* genotype *in vivo*. The resulting segregation of cases according to genotype was associated with likelihood of responsiveness. However, there were cases lacking the genetic background predictive of susceptibility to therapy that still did not respond to the treatment. This is a classic example of the multifactorial road to immune responsiveness [29] underlining that multiple factors may be necessary to allow response to therapy, among which the host genetic background is one. Thus, it is possible that this separate set of genes has distinct yet complementary influences on immune responsiveness.

Fine mapping studies now underway suggest that there are two independent associations with *IRF5* defining risk for lupus (L. Kottyan, J. Harley, K.M. Kaufman, unpublished data). The markers identified as being associated with the responses to TILs in this study are more associated with the association located in the promoter of *IRF5*. In addition, in lupus there are suggestive data that sub-phenotypes also have variable associations with *IRF5* alleles [30], suggesting rich variation in the *IRF5* control of immune responsiveness.

As a theoretical limitation of this study, we refrained from genotyping the melanoma metastases with the assumption that polymorphisms of the germline were sufficiently representative. Due to the genetic instability of the tumor genome, genotyping the tumor DNA instead of the germline DNA would be ideal for association studies. However, tumor tissue samples are composed of heterogeneous cell types and it is not clear which one mostly contributes to immune responsiveness. Thus, this study was aimed only at the predictive effects of the genetic background of the host. On the other hand, comparison of 15 cell lines with their parental tumors suggested that in the large majority of cases the *IRF5* gene was conserved. The frequency of LOH was < 10%, which is close to other estimates of LOH prevalence for genes not directly related to oncogenesis such as the human leukocyte antigen class I genes [31-33]. Thus, we are confident that this analysis approximates the results that could be obtained if the analysis could have been adjusted according to potential somatic changes in the *IRF5* gene.

In conclusion, polymorphism of *IRF-5* appears to be a predictor of immune responsiveness of melanoma metastases to adoptive therapy with TILs. Comparison of melanoma cell lines classified according to the AA vs. GG rs10954213 (G > A) suggested significant differences in global transcription enriched in genes related to immune regulation. The signatures differentiating the 2 cell line genotypes *in vitro* are predictive of the responsiveness of melanoma metastases *in vivo*. Thus, it appears that immune responsiveness is at least in part dependent on the genetic background of the host, which affects the biology of cancer cells primarily and secondarily the immune responsiveness of tumors. This is the first study analyzing the role of *IRF5* gene polymorphism in determining immune responsiveness of melanoma. The results provide a link between the determinism of autoimmunity and immune responsiveness and suggest a potential genetic marker that could be evaluated prospectively and eventually considered for future patient stratification.

## Competing interests

The authors declare that they have no competing interests.

## Authors’ contribution

FMM LU conceived the study, LU performed the experiments. LU YZ VDG FM analyzed the data. LU and FM drafted the manuscript, JHB KMK LK revised data and manuscript. BT NE MED ST DB QL MLA RS WE SAR revised critically the manuscript for important intellectual content and for the language. All the authors read and approved the final manuscript.

## Supplementary Material

Additional file 1 **Table S1. **Table of significant genes for testing 'genotype' effect (106 genes were found significant at level 0.01). Click here for file
