# Supplementary material for: IRF5 gene polymorphisms in melanoma
Source: J Transl Med. 2012 Aug 21;10:170. doi: 10.1186/1479-5876-10-170 (PMC3492128; doi:10.1186/1479-5876-10-170)
Supplement: Additional file 1 — Table S1. Table of significant genes for testing 'genotype' effect (106 genes were found significant at level 0.01). [file 1479-5876-10-170-S1.doc]

**Supplementary tables**

Table S1. Table of significant genes for testing 'genotype' effect (106 genes were found significant at level 0.01).

|  | UniqueID | Annotations | Symbol | Defined Genelists P-value |
| --- | --- | --- | --- | --- |
| 1 | 8019954 | FLJ35776 |  | 0 |
| 2 | 8150698 | SNAI2 | Adherens junction | 0.0001 |
| 3 | 7978553 | C14orf147 |  | 0.0003 |
| 4 | 8152764 | MTSS1 | Perou's- Intrinsic- Breast-Cancer-Genes | 0.0004 |
| 5 | 7901175 | TSPAN1 |  | 0.0004 |
| 6 | 7929634 | ZDHHC16 |  | 0.0007 |
| 7 | 8033097 | RFX2 | immunology, transcription | 0.001 |
| 8 | 7899284 | TRNP1 |  | 0.001 |
| 9 | 8026365 | ZNF333 |  | 0.0011 |
| 10 | 7958152 | PGAM1 | misc | 0.0013 |
| 11 | 7930162 | C10orf26 |  | 0.0013 |
| 12 | 7903162 | TMEM56 |  | 0.0014 |
| 13 | 8007707 | CCDC103 |  | 0.0014 |
| 14 | 8043835 | C2orf15 |  | 0.0015 |
| 15 | 7898405 |  |  | 0.0017 |
| 16 | 7912806 |  |  | 0.0017 |
| 17 | 7944722 | UBASH3B |  | 0.0018 |
| 18 | 8025328 | EVI5L |  | 0.0018 |
| 19 | 7971590 | CAB39L |  | 0.002 |
| 20 | 8004030 | RNF167 |  | 0.002 |
| 21 | 7936064 | NT5C2 | Nicotinate and nicotinamide metabolism, Purine metabolism, Pyrimidine metabolism | 0.002 |
| 22 | 7990987 | AP3B2 |  | 0.0021 |
| 23 | 7935746 | BLOC1S2 |  | 0.0023 |
| 24 | 7928695 | C10orf58 |  | 0.0023 |
| 25 | 8099364 | ZNF518B |  | 0.0025 |
| 26 | 7998457 | UNKL |  | 0.0026 |
| 27 | 7934299 | NUDT13 |  | 0.0027 |
| 28 | 8164105 | GOLGA1 |  | 0.0029 |
| 29 | 8124940 | SNORD117 |  | 0.0029 |
| 30 | 7965480 | PGAM1 | misc | 0.003 |
| 31 | 7936242 | ITPRIP |  | 0.0031 |
| 32 | 7919600 |  |  | 0.0033 |
| 33 | 7928736 | NRG3 | g-Secretase mediated ErbB4 Signaling Pathway | 0.0033 |
| 34 | 7929624 | PGAM1 | misc | 0.0033 |
| 35 | 7975772 | FAM164C |  | 0.0034 |
| 36 | 8134351 | PPP1R9A |  | 0.0034 |
| 37 | 7998233 | TMEM8A |  | 0.004 |
| 38 | 7930148 | SFXN2 |  | 0.004 |
| 39 | 7919578 | GSTM2 | Perou's- Intrinsic- Breast-Cancer-Genes, Glutathione metabolism, Metabolism of xenobiotics by cytochrome P450, immunology, pharmacology | 0.0041 |
| 40 | 7931216 | FAM175B |  | 0.0041 |
| 41 | 8177867 | DDR1 |  | 0.0041 |
| 42 | 8113083 | FAM172A |  | 0.0042 |
| 43 | 7912537 | DHRS3 | 1- and 2-Methylnaphthalene degradation, Benzoate degradation via CoA ligation, Bisphenol A degradation, Ethylbenzene degradation, gamma- Hexachlorocyclohexane degradation, Limonene and pinene degradation | 0.0043 |
| 44 | 7935425 | RRP12 |  | 0.0044 |
| 45 | 8157828 | ARPC5L | Regulation of actin cytoskeleton | 0.0044 |
| 46 | 8088397 | ACOX2 | Perou's- Intrinsic- Breast-Cancer-Genes, Fatty acid metabolism, PPAR signaling pathway | 0.0045 |
| 47 | 7980680 | FOXN3 |  | 0.0045 |
| 48 | 8111129 | ZNF622 |  | 0.0047 |
| 49 | 8111136 | FAM134B |  | 0.0047 |
| 50 | 8120067 | SLC25A27 |  | 0.0048 |
| 51 | 8173729 | PGAM4 | Glycolysis / Gluconeogenesis | 0.0048 |
| 52 | 8176282 | PGAM4 | Glycolysis / Gluconeogenesis | 0.0048 |
| 53 | 8100603 | CENPC1 | DNA_replication, immunology | 0.005 |
| 54 | 8157144 | C9orf6 |  | 0.0051 |
| 55 | 7956867 | HMGA2 |  | 0.0051 |
| 56 | 8165735 | CSF2RA | Cytokine-cytokine receptor interaction, Hematopoietic cell lineage, Jak-STAT signaling pathway, immunology | 0.0053 |
| 57 | 8176306 | CSF2RA | Cytokine-cytokine receptor interaction, Hematopoietic cell lineage, Jak-STAT signaling pathway, immunology | 0.0053 |
| 58 | 7927267 | FAM35B | mTOR Signaling Pathway, Insulin signaling pathway, mTOR signaling pathway | 0.0054 |
| 59 | 8067033 | KCNG1 |  | 0.006 |
| 60 | 7930120 | SUFU | Sonic Hedgehog (Shh) Pathway, Hedgehog signaling pathway | 0.0061 |
| 61 | 8068593 | ETS2 | METS affect on Macrophage Differentiation, Dorsoventral axis formation, transcription, tsonc | 0.0064 |
| 62 | 8117640 | ZSCAN16 |  | 0.0064 |
| 63 | 8100941 |  |  | 0.0064 |
| 64 | 8179184 | DDR1 |  | 0.0065 |
| 65 | 8155563 | MTHFD1L | Glyoxylate and dicarboxylate metabolism, One carbon pool by folate | 0.0065 |
| 66 | 8125530 | HLA-DMB | Antigen processing and presentation, Cell adhesion molecules (CAMs), Type I diabetes mellitus, immunology | 0.0066 |
| 67 | 8139820 | ZNF680 |  | 0.0066 |
| 68 | 8010243 | SYNGR2 |  | 0.0067 |
| 69 | 7927288 | FAM35B2 |  | 0.0068 |
| 70 | 8161484 | MTHFD1L | Glyoxylate and dicarboxylate metabolism, One carbon pool by folate | 0.0068 |
| 71 | 8042283 | HSPC159 |  | 0.007 |
| 72 | 7935462 | EXOSC1 |  | 0.007 |
| 73 | 8117435 | BTN3A2 |  | 0.0071 |
| 74 | 7912638 | C1orf126 |  | 0.0071 |
| 75 | 8160712 | SUGT1P1 |  | 0.0072 |
| 76 | 8122457 | STX11 | SNARE interactions in vesicular transport | 0.0073 |
| 77 | 8173551 | PHKA1 | Calcium signaling pathway, Insulin signaling pathway | 0.0074 |
| 78 | 7935730 | CWF19L1 |  | 0.0074 |
| 79 | 8148548 | PSCA |  | 0.0074 |
| 80 | 7928909 | FAM35A |  | 0.0074 |
| 81 | 7898663 | PINK1 | Neurodegenerative Disorders, Parkinson\'s disease | 0.0074 |
| 82 | 7935819 | MRPL43 |  | 0.0075 |
| 83 | 7917779 | GCLM | Glutamate metabolism, Glutathione metabolism | 0.0075 |
| 84 | 8076962 | MAPK12 | MAPKinase Signaling Pathway, Epithelial cell signaling in Helicobacter pylori infection, Fc epsilon RI signaling pathway, GnRH signaling pathway, Leukocyte transendothelial migration, MAPK signaling pathway,Toll-like receptor signaling pathway, VEGF signaling pathway | 0.0076 |
| 85 | 8007493 | ARL4D |  | 0.0077 |
| 86 | 8166184 | CA5B | Nitrogen metabolism | 0.0078 |
| 87 | 7930226 | PDCD11 |  | 0.0078 |
| 88 | 8180078 | HLA-DMB | Antigen processing and presentation, Cell adhesion molecules (CAMs), Type I diabetes mellitus, immunology | 0.0079 |
| 89 | 7938012 | TRIM6- |  | 0.0082 |
| 90 | 8164314 | ST6GALNAC4 | Glycan structures - biosynthesis 2, Glycosphingolipid biosynthesis - ganglioseries | 0.0082 |
| 91 | 7978407 | PRKD1 |  | 0.0083 |
| 92 | 7963923 | SARNP |  | 0.0084 |
| 93 | 8124397 | HIST1H1C | Perou's- Intrinsic- Breast-Cancer-Genes | 0.0085 |
| 94 | 7940160 | DTX4 | Notch signaling pathway | 0.0086 |
| 95 | 8165024 | C9orf116 |  | 0.0086 |
| 96 | 7978911 | C14orf182 |  | 0.0086 |
| 97 | 8159379 | TMEM141 |  | 0.0089 |
| 98 | 7996647 | PARD6A | Tight junction | 0.0094 |
| 99 | 7918620 | FLJ36116 |  | 0.0094 |
| 100 | 7933084 | NAMPT | Nicotinate and nicotinamide metabolism | 0.0095 |
| 101 | 7913593 | TCEA3 | Perou's- Intrinsic- Breast-Cancer-Genes | 0.0095 |
| 102 | 8074157 | RPL23AP82 |  | 0.0098 |
| 103 | 7965964 | SLC41A2 |  | 0.0099 |
| 104 | 7965335 | DUSP6 | Regulation of MAP Kinase Pathways Through Dual Specificity Phosphatases, MAPK signaling pathway | 0.0099 |
| 105 | 8067029 | KCNG1 |  | 0.0099 |
| 106 | 7904693 |  |  | 0.0099 |
